# Global shockwaves of the Hunga Tonga-Hunga Ha’apai volcano eruption measured at ground stations

**DOI:** 10.1016/j.isci.2022.105356

**Published:** 2022-10-13

**Authors:** Chunyan Li

**Affiliations:** 1 Department of Oceanography and Coastal Sciences, College of the Coast and Environment, Louisiana State University, Baton Rouge, LA 70803, USA; 2 Coastal Studies Institute, Louisiana State University, Baton Rouge, LA 70803, USA

**Keywords:** Earth sciences, Geology, Volcano, Atmospheric observation

## Abstract

The eruption of the Tonga volcano created globally propagating spherical shockwaves in the atmosphere. Analyses are done to data from two southern U.S. stations of the author sampling at 3–21 s intervals and 189 weather stations at 1–5 min intervals. The shockwaves arrived from two routes in the atmosphere: the shortest spherical arc and the longer spherical arc through the antipole. In most stations, signals up to the 6^th^ path of shockwaves were recorded as the waves traveled around the globe multiple times. The speed of shockwaves is estimated to be 309.5 ± 2.9 m/s, consistent with the speed of sound at the top of the troposphere where a waveguide exists. Discussion is made on the post-shockwave ringing of 4–8 min as higher amplitude oscillations above the level of pre-shockwaves background noise. A theoretical wave dispersion is derived which verifies that the spherical shockwave’s phase speed is the same as the speed of sound.

## Highlights

• Dispersion equation of global spherical shockwaves from a point source derived
• Air pressure of 3–21 s recorded the shockwaves from the Tonga volcano eruption
• Shockwaves and ringing with higher energy analyzed using data from 191 stations
• Shockwaves up to 6^th^ pass from 191 stations consistent with global spherical waves

## Graphical abstract

**Figure:**
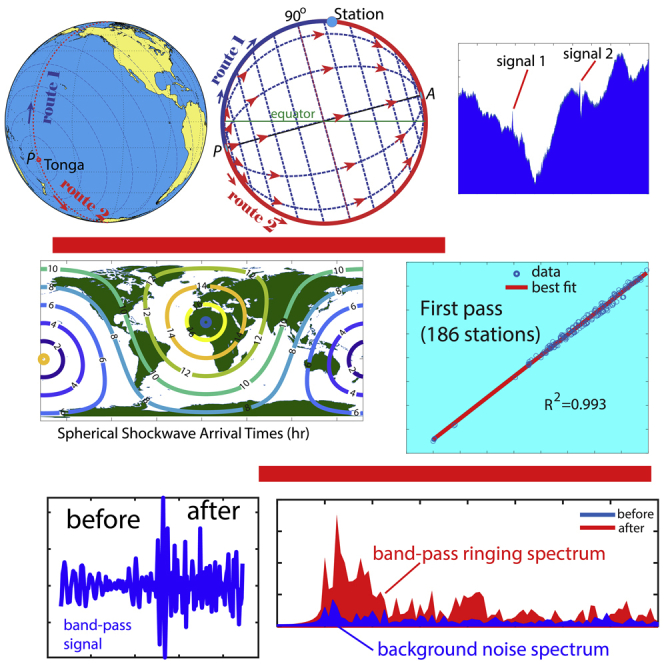

## Introduction

**Figure 1:**
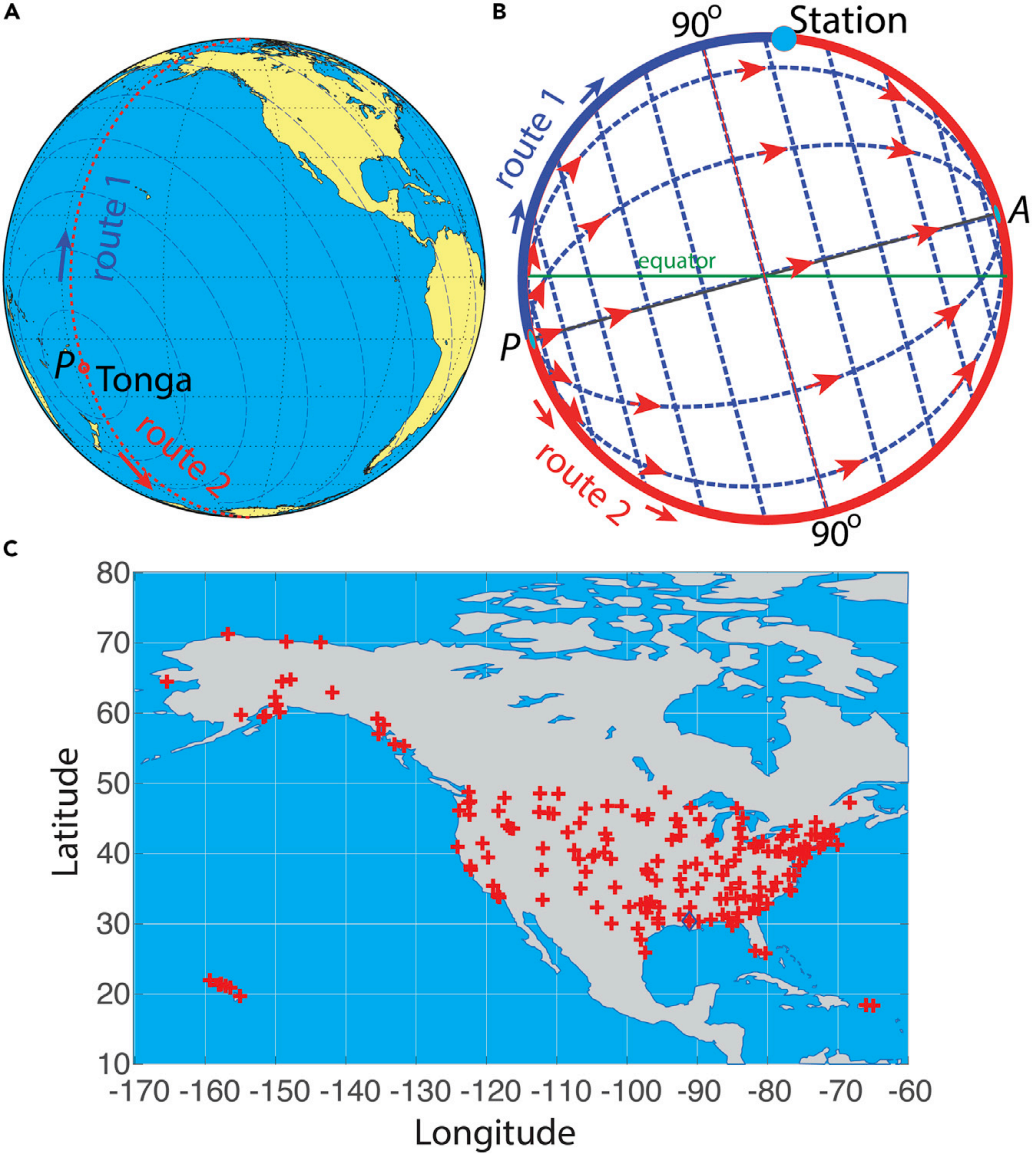
Study site and shockwave routes The Hunga Tonga-Hunga Ha’apai underwater volcano site P in the southern Pacific at (20°33′0.00"S, 175°23′6.00"W), near the International Date Line (approximately along the red dashed line in (A)). In (B), point *A* is the antipole at (20°33′0.00"N, 4°36′54"E). The green line is the equator. The thin red line indicates the great circle of 90° from *P* and the red arrows indicate the directions of the shockwave propagations. The blue arc shows Route 1, the shortest arc on the great circle to reach the sampling site marked as Station. The red arc is Route 2, the longer arc on the great circle to reach S through the antipole *A*. The stations are shown in (C), except the stations at Guam (Figure 8). The diamond shows the stations in Louisiana where high-resolution data (with 3-s and 21-s intervals) are collected.

On 15 January2022, a catastrophic eruption of the Hunga Tonga-Hunga Ha’apai underwater volcano in the southern Pacific Ocean (Figure 1A) occurred after 0400 UTC (Terry et al., 2022). It was reported by the World Bank (2022) that the eruption occurred at 04:14:45 UTC (17:14:45, 15 January, local time). Yuen et al. (2022) reported that the eruption started at 0402 UTC and drastically increased at 0408 UTC; others reported that it started at 0415 UTC (Burt, 2022), or before 0410 UTC (Smart, 2022) according to the satellite images (Bachmeier, 2022). This volcano was in the southern Pacific at (20.55° S, 175.385°W), approximately 1900 km north-northeast of New Zealand, 3200 km east of Australia, and 600 km southeast of Fiji. The nearby islands of Hunga Tonga and Hunga Ha’apai with an area of 1 km^2^ and a maximum altitude of 149 m were blown apart. Eruption-induced tsunami waves were observed in the Pacific Ocean, damaging at least 600 structures including 300 residential buildings. The economic damage was estimated greater than 90M US dollars (World Bank, 2022). The explosion is believed to be a once-in-1000-year’s event for the Hunga caldera. The eruption reached at least 35- to 45-km altitude (Bachmeier, 2022) and transiently 58 km (Yuen et al., 2022). It created what appeared to be spherical shockwaves propagating around the globe (Smart, 2022; Amores et al., 2022), as captured by the geostationary satellites including NOAA’s GEOS-17 and Japanese Himawari-8 (Bachmeier, 2022), and by barometric pressure at ground stations up to 127 h measured in Britain and Ireland (Burt, 2022). The eruption energy (4–18 megatons, pending confirmation, World Bank, 2022) was estimated to be below that of the explosion at Mount St. Helens (US) in 1980 (with 24 megatons of energy) and the Krakatoa (Indonesia) explosion in 1883 (200 megatons of energy, Symons, 1888; Yokoyama, 1981; Gabrielson, 2010). The 1883 Krakatoa eruption (Symons, 1888; Gabrielson, 2010) produced globally propagating shockwaves lasting for at least 5 days. Preliminary analysis puts the Tonga eruption as the largest volcanic eruption of this century, and since 1991, the Mount Pinatubo eruption (Smith and Kilburn, 2010). On a scale of 0–8 for the volcanic explosive index (VEI), the Hunga Tonga-Hunga Ha’apai underwater volcano eruption is tentatively ranked at 5 (World Bank, 2022), indicating that the total volume ejection was greater than 1 km^3^. These assessments, however, are preliminary and may be updated as studies continue.

Explosive volcanic eruptions typically generate shockwaves (Nairn, 1976), acoustic waves (Woulff and McGetchin, 1958) including infrasound waves propagating in the atmosphere (Morrissey and Chouet, 1997) detectable by microbarographs and electric signals in the atmosphere measurable by lightning mapping array (LMA) systems. Earthquakes can also produce infrasound waves measurable in the atmosphere (Shani-Kadmiel et al., 2021). Seismic waves from volcanic eruptions transmit through the ground whereas infrasound waves from volcanic eruptions propagate in the atmosphere (Smith et al., 2020). It has been known for a long time that volcanic eruptions can produce measurable atmospheric pressure waves (Yokoo et al., 2006) at weather stations, and air pressure variations have been used to estimate the total energy from the eruptions (Gorshkov, 1960).

Atmospheric (barometric) pressure data from meteorological stations and data from infrasound sensors are useful for studying eruption/explosion (including manmade explosions especially nuclear explosions, e.g., Perttu et al., 2020; Pichon et al., 2019). Early observations and studies of infrasound and atmospheric waves caused by volcanic eruptions used analog instruments. Data from different stations can be combined to make inferences about the events. Using such data, some parameters of a mysterious Siberian explosion in 1908 were determined (Ben-Menahem, 1975): the atmospheric shockwave was propagating at a speed of 285–324 m/s, and the total energy was estimated at ∼ 12.5 ± 2.5 megatons (1 megaton = 4.18 × 10^15^ joules) from an unknown source. The difference in the wave speed estimates was believed to be caused by the different meteorological conditions at different stations. Another study analyzing eruption movies concluded that the pressure waves induced by the eruptions had phase speeds ranging between 342 m/s and 574 m/s (Yokoo and Ishihara, 2007).

Mathematical models have been developed to study explosion processes especially near-field dynamics, to quantify motions and wave propagations (Clarke et al., 2002). Shockwaves from volcanic eruptions have been simulated using 3D numerical models for near-field dynamics (Saito and Takayama, 2005). In an early article (Pekeris, 1939), a mathematical solution from Lamb (1932) was used to study sound wave propagation in the atmosphere due to a disturbance that is applicable to volcanic eruptions, although the model ignored the curvature of the Earth (not spherical waves) when considering wave propagation in the atmosphere. According to numerical model computations, the exit velocity during a volcanic eruption can be as high as 300 m/s, close to the speed of sound (Turcotte et al., 1990). Another study using a mathematical model showed that when a volcano erupts, the exit silicic magma and volatiles can increase the air pressure by 10–100 times of the atmospheric pressure (Woods and Bower, 1995). The volcanic eruption exit speed rapidly decreases as the materials exiting the volcano are decompressed with reduced pressure in the air but at 0–1 km above the crater rim, the velocity can still be comparable to the speed of sound or even supersonic (Self et al., 1979). Large volcano explosions can release a tremendous amount of energy which generates atmospheric waves and infrasound waves traveling on global scales and the signals can be detected more than 10,000 km away (Dabrowa et al., 2011).

Air pressure data from meteorological stations (Automated Surface Observing Systems, or ASOS) in the U.S. and similar systems elsewhere typically report data at 1-min, 5-min to 1-h intervals. The hourly data are too sparse to capture shockwave events. The 5-min interval data are marginally useful if used alone. When these data are combined with high-resolution data; however, they can corroborate the findings. In this article, we report the use of data from air pressure sensors sampling at 3 and 21 s intervals, making it possible to resolve the signals without aliasing (Proakis and Manolakis, 1992), allowing an accurate computation of the arrival times of the maximum disturbance, the evolution of the signal, and the propagation speed of the waves. In addition, we also use 1-min and 5-min interval data from 189 additional weather stations (Figure 1 and Tables 1, 2, 3, and 4). When data from multiple stations are combined, it allows us to have a holistic view of the shockwaves to reconstruct a global picture of the shockwaves traveling around our planet. We have already seen publications reporting findings about the characteristics of shockwaves and wave propagation speed (Burt, 2022; Harrison, 2022) and other related physical processes (Yuen et al., 2022). In this study, we extend the study to cover different regions with a combination of high (3-s to 1-min) and low (5-min) temporal resolutions. In addition, we also provide a theoretical analysis of the spherical shockwaves in terms of the dispersion relationship.

**Table 1:** Locations of Stations. No. 1 & 2 are sites with high-resolution data; No. 3–40 are sites with 5-min data

No.	Name	Longitude	Latitude	Distance from Tonga Volcano (km)
1	Ridge, Louisiana	−91.0912	30.3695	10627
2	Russell, Louisiana	−91.1794	30.4116	10621
3	O'Hare Airport, Chicago,	−87.9319	41.9875	11316
4	San Francisco, California	−122.2207	37.7213	8534
5	Baton Rouge, Louisiana	−91.1469	30.5372	10629
6	Boston, Massachusetts	−71.0097	42.3606	12687
7	Phoenix, Arizona	−112.0116	33.4343	9004
8	Tacoma Airport, Seattle	−122.3144	47.4447	9229
9	Dickinson, North Dakota	−102.8019	46.7973	10416
10	Westhampton Beach, Long Island	−72.6318	40.8436	12532
11	Norfolk, Virginia	−76.1922	36.9033	12153
12	Newark, New Jersey	−74.1693	40.6827	12401
13	Ann Arbor, Michigan	−83.7397	42.224	11656
14	Barrow, Alaska	−156.7922	71.2826	10311
15	Los Angeles, California	−118.3865	33.9382	8548
16	Savannah, Georgia	−81.2021	32.1276	11581
17	Groton, Connecticut	−72.05	41.33	12588
18	Adak Island, Alaska	−176.646	51.878	8055
19	Anchorage, Alaska	−149.8573	61.2163	9375
20	Barter Island, Alaska	−143.5819	70.134	10388
21	Mobile, Alabama	−88.0681	30.6268	10910
22	Little Rock, Arkansas	−92.2357	34.7273	10698
23	Denver, Colorado	−104.6575	39.8328	9928
24	Dallas, Texas	−96.8518	32.8471	10224
25	Honolulu, Hawaii	−157.9224	21.3187	5027
26	Caldwell, Idaho	−116.6358	43.6419	9310
27	Miami, Florida	−80.3169	25.788	11468
28	San Antonio, Texas	−98.4711	29.337	9926
29	Brownsville, Texas	−97.4231	25.9146	9866
30	Memphis, Tennessee	−89.985	35.0611	10904
31	Salt Lake City, Utah	−111.97	40.78	9446
32	Ithaca, New York	−76.4584	42.491	12247
33	Raleigh, North Carolina	−78.7819	35.8922	11902
34	Reno, Nevada	−119.7711	39.4839	8822
35	Riverton, Wyoming	−108.4598	43.0642	9826
36	Havre, Montana	−109.7633	48.5428	10053
37	St. Paul, Minnesota	−93.06	44.9345	11027
38	Salisbury, Maryland	−75.5103	38.3405	12243
39	Frenchville, Maine	−68.3127	47.2855	12941
40	Sanford, Maine	−70.708	43.3939	12724

**Table 2:** Locations of Stations with 1-min data (Part I). Shown here are the names, coordinates and distances

No.	Name	Longitude	Latitude	Distance from Tonga Volcano (km)
41	TJSJ	−66.0021	18.4394	12670
42	TIST	−64.9733	18.3373	12772
43	PHTO	−155.0485	19.7203	4995
44	PHOG	−156.4305	20.8986	5049
45	PHNL	−157.9202	21.3178	5027
46	PHNG	−157.7679	21.4505	5047
47	PHMK	−157.0963	21.1529	5045
48	PHLI	−159.3390	21.9760	5039
49	PHJR	−158.0703	21.3074	5020
50	PGUM	144.7971	13.4840	5767
51	PGSN	145.7300	15.1202	5811
52	PAWD	−149.4166	60.1299	9274
53	PATK	−150.0927	62.3214	9482
54	PASO	−151.7050	59.4439	9153
55	PASI	−135.3611	57.0468	9398
56	PASC	−148.4652	70.1948	10310
57	PAOR	−141.9281	62.9612	9737
58	PAOM	−165.4444	64.5126	9497
59	PANN	−149.0739	64.5473	9729
60	PANC	−149.9981	61.1741	9367
61	PAMR	−149.8447	61.2135	9375
62	PAKW	−133.0760	55.5792	9357
63	PAKT	−131.7112	55.3541	9393
64	PAJN	−134.5785	58.3547	9542
65	PAIL	−154.9178	59.7556	9122
66	PAHO	−151.4858	59.6450	9179
67	PAHN	−135.5235	59.2438	9587
68	PAFA	−147.8567	64.8154	9780
69	PABR	−156.7686	71.2849	10312
70	KWLD	−97.0375	37.1686	10399
71	KVIH	−91.7695	38.1274	10869
72	KVAY	−74.8457	39.9429	12331
73	KUTS	−95.5872	30.7469	10243
74	KTYS	−83.9941	35.8111	11446
75	KTYR	−95.4030	32.3535	10328
76	KTVR	−91.0277	32.3516	10710
77	KTUL	−95.8881	36.1984	10451
78	KTTD	−122.4013	45.5494	9086
79	KTLH	−84.3509	30.3968	11240
80	KTKI	−96.5888	33.1771	10261
81	KTIW	−122.5781	47.2679	9201
82	KTHV	−76.8730	39.9170	12161
83	KSDF	−85.7365	38.1741	11371
84	KRST	−92.5000	43.9083	11031
85	KPAH	−88.7730	37.0603	11079
86	KORH	−71.8756	42.2671	12615
87	KONO	−117.0130	44.0194	9311
88	KNKT	−76.8808	34.9032	12045
89	KMWL	−98.0602	32.7816	10118
90	KMRH	−76.6604	34.7338	12061
91	KMLS	−105.8882	46.4269	10188

∗Note: The stations with their names starting with T are those in the tropical Atlantic; those with P are the Pacific stations (Hawaii, Alaska, and Guam); and those starting with K are those in the contiguous U.S.

**Table 3:** Locations and Distance of Stations with 1-min data (Part II). Shown here are the names, coordinates and distances

No.	Name	Longitude	Latitude	Distance from Tonga Volcano (km)
92	KMIA	−80.2901	25.7954	11471
93	KMEB	−79.3659	34.7922	11822
94	KMBS	−84.0796	43.5329	11665
95	KLVM	−110.4480	45.6994	9843
96	KLNS	−76.2944	40.1224	12213
97	KLGB	−118.1519	33.8179	8557
98	KJST	−78.8347	40.3156	12006
99	KITR	−102.2854	39.2425	10079
100	KHZY	−80.6968	41.7778	11888
101	KHUF	−87.3070	39.4506	11283
102	KHHR	−118.3351	33.9229	8551
103	KFTW	−97.3624	32.8198	10179
104	KFOE	−95.6636	38.9509	10586
105	KELZ	−77.9900	42.1095	12115
106	KDWH	−95.5528	30.0618	10216
107	KDSV	−77.7133	42.5705	12147
108	KDEW	−117.4286	47.9671	9549
109	KDAW	−70.9295	43.2842	12705
110	KCVG	−84.6678	39.0488	11488
111	KCUB	−80.9952	33.9705	11654
112	KCTB	−112.3762	48.6084	9895
113	KCPS	−90.1551	38.5704	11018
114	KCNM	−104.2634	32.3374	9573
115	KCLE	−81.8547	41.4094	11785
116	KCHS	−80.0405	32.8986	11709
117	KCHA	−85.2036	35.0352	11316
118	KCDR	−103.0954	42.8376	10197
119	KCAG	−107.5217	40.4952	9750
120	KBZN	−111.1503	45.7772	9802
121	KBYG	−106.7218	44.3811	10020
122	KBWG	−86.4197	36.9645	11275
123	KBUR	−118.3587	34.2007	8567
124	KBTV	−73.1533	44.4720	12543
125	KBTM	−112.4975	45.9548	9725
126	KBPK	−92.4705	36.3689	10743
127	KBOI	−116.2229	43.5644	9332
128	KBLI	−122.5375	48.7927	9315
129	KBLF	−81.2075	37.2959	11729
130	KBJJ	−81.8882	40.8748	11769
131	KBIS	−100.7457	46.7727	10555
132	KBHM	−86.7523	33.5639	11131
133	KBFL	−119.0577	35.4339	8598
134	KBDE	−94.6111	48.7302	11062
135	KBCE	−112.1458	37.7064	9248
136	KAUW	−89.6270	44.9263	11283
137	KATY	−97.1547	44.9140	10726
138	KATL	−84.4279	33.6367	11339
139	KASX	−90.9190	46.5485	11243
140	KAST	−123.8786	46.1580	9045
141	KASE	−106.8682	39.2219	9729

**Table 4:** Locations and Distance of Stations with 1-min data (Part III). Shown here are the names, coordinates and distances

No.	Name	Longitude	Latitude	Distance from Tonga Volcano (km)
142	KASD	−89.8208	30.3463	10741
143	KART	−76.0194	43.9918	12309
144	KARR	−88.4757	41.7719	11266
145	KARB	−83.7457	42.2229	11655
146	KAQW	−73.1706	42.6963	12516
147	KAPN	−83.5603	45.0781	11747
148	KAPF	−81.7756	26.1524	11340
149	KAPC	−122.2807	38.2132	8565
150	KAPA	−104.8493	39.5701	9900
151	KAOO	−78.3200	40.2964	12048
152	KAOH	−84.0271	40.7075	11590
153	KANJ	−84.3684	46.4792	11723
154	KAND	−85.8581	33.5882	11211
155	KANB	−85.8581	33.5882	11211
156	KAMW	−93.6218	41.9920	10871
157	KAMG	−82.5066	31.5361	11445
158	KAMA	−101.7059	35.2194	9928
159	KALW	−118.2841	46.0925	9371
160	KALS	−105.8679	37.4351	9709
161	KALO	−92.4010	42.5584	10987
162	KALI	−98.0269	27.7409	9893
163	KALB	−73.8020	42.7491	12466
164	KAKR	−81.4669	41.0375	11807
165	KAKQ	−77.0011	36.9872	12084
166	KAKO	−103.2220	40.1756	10054
167	KAKH	−81.1499	35.2026	11676
168	KAIA	−102.8037	42.0532	10180
169	KAHN	−83.3259	33.9486	11447
170	KAGS	−81.9645	33.3699	11550
171	KAGC	−79.9290	40.3544	11916
172	KAFW	−97.3194	32.9904	10191
173	KAFN	−72.0030	42.8051	12612
174	KAEX	−92.5486	31.3274	10535
175	KACY	−74.5772	39.4576	12345
176	KACV	−124.1085	40.9778	8646
177	KACT	−97.2303	31.6122	10137
178	KACK	−70.0599	41.2530	12752
179	KABY	−84.1945	31.5355	11292
180	KABR	−98.4224	45.4468	10658
181	KABQ	−106.6083	35.0389	9523
182	KABI	−99.6819	32.4113	9963
183	KABE	−75.4404	40.6524	12295
184	KAAT	−120.5654	41.4829	8909
185	KAAO	−97.2211	37.7476	10409
186	KAAF	−85.0274	29.7275	11155
187	K79J	−86.3922	31.3084	11085
188	K12N	−74.7380	41.0086	12360
189	K8D3	−96.9936	45.6695	10770
190	K6R6	−102.2132	30.0465	9632
191	K1J0	−85.6017	30.8439	11141

## Results

### The time series and signals of shockwaves at Sites 1 and 2

**Figure 2:**
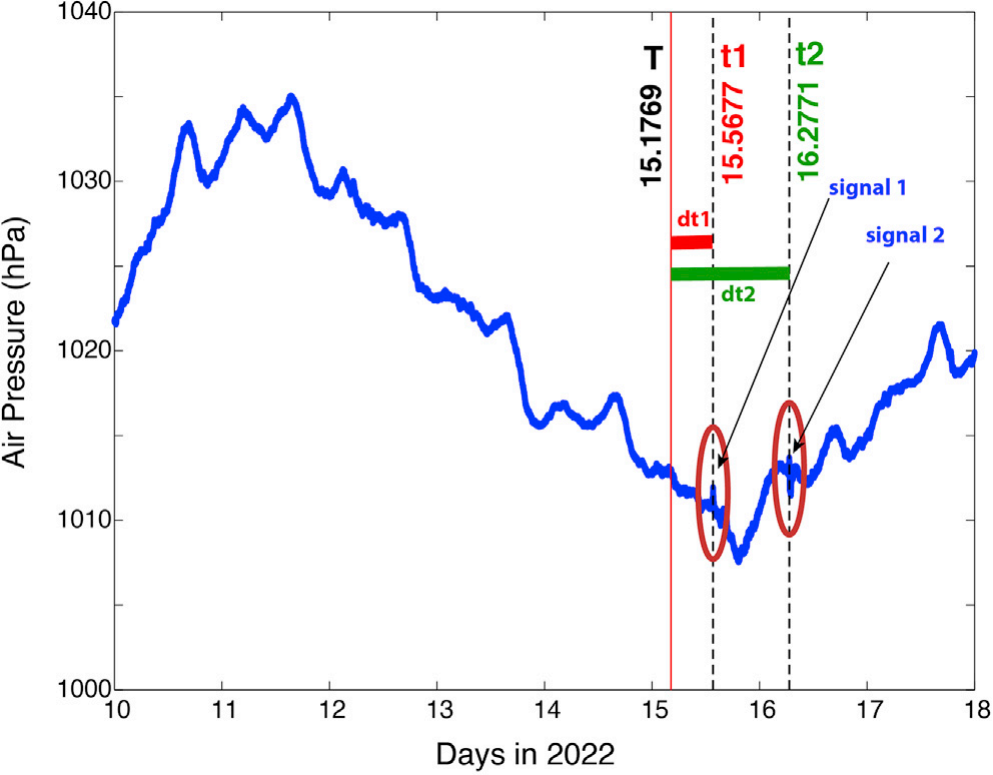
Shockwave signals from the two routes Time series of air pressure at Station 1 (Table 1) between 10 and 18 January 2022 UTC. The red line indicates the time (T) of the major eruption that produced the air pressure fluctuations propagated globally. The dashed green lines indicate the times with sharp fluctuations of pressure, corresponding to the first and second arrivals of the shockwave signal through Routes 1 and 2 (Figure 1), respectively.

**Figure 3:**
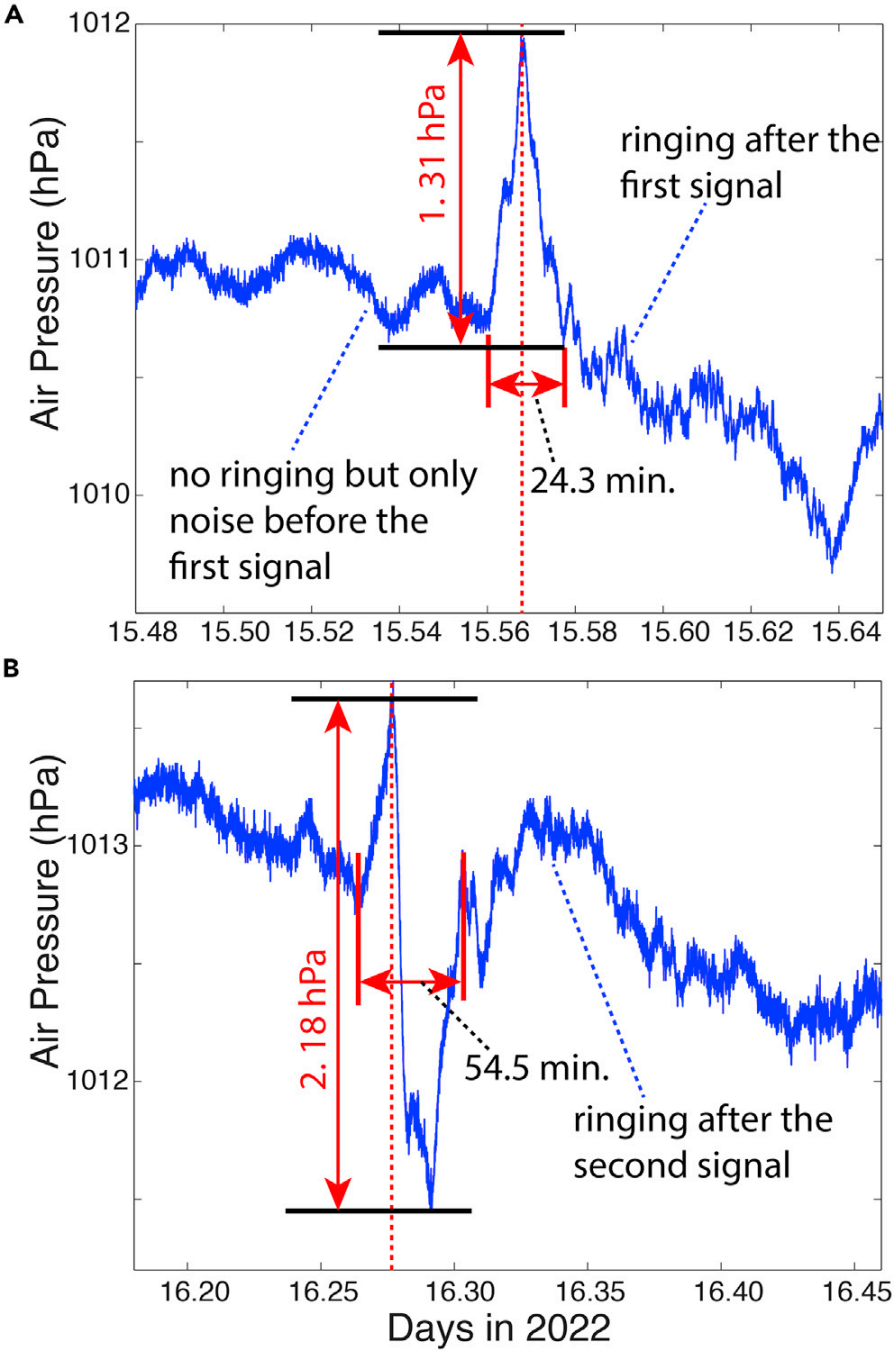
Zoomed in view of shockwave signals from the high-resolution pressure data Time series of air pressure from Site 1 (Table 1). The top panel (A) covers the period of the first shockwave signal through Route 1 (Figure 1), and bottom panel (B) covers the period of the second signal through Route 2. Data from Site 2 are similar.

The high-resolution observations at 3–21 s intervals of air pressure from the author’s two stations show two abrupt variations (the circled fluctuations in Figure 2) after the catastrophic eruption on 15 January. Although there are fluctuations in the time series data, these two peaks are unique such that they are sharp (short period) and relatively large, making them stand out. The first signal shows an abrupt increase in air pressure of approximately 1.31 hPa in ∼12 min, followed by a rapid decrease at about the same rate. The duration of the first signal is approximately 24.3 min (Figure 3A). The second signal (Figure 3B) has a similar rapid increase but with a smaller magnitude (∼0.9 hPa), followed by a rapid drop in pressure by 2.18 hPa. The duration of the second signal is longer (54.5 min, Figure 3B). It is noted that after each of the signals, there appears to be some “ringing”: waves at higher frequencies (∼ a few minutes in period).

The arrival time of the first signal at Site 1 was ∼13:37:26 UTC on 15 Jan., or 9 h, 22 min, and 41 s (or 9.378 h, Table 5) after the eruption. If the peak of the signal corresponds to the maximum eruption, given the distance between P and Site 1 of 10627 km (Table 1), the propagation speed of the wave is estimated at 314.8 m/s. This is consistent with the shockwave speed reported in previous studies (e.g., Ben-Menahem, 1975) and recent reports for the Tonga event (306–315 m/s by Harrison, 2022).

**Table 5:** Arrival time of shockwaves, time of travel, and propagation speed. No. 1 & 2 are from the high-resolution stations; No. 3–40 are for those stations with data sampled at 5-min intervals

Station No.	Arrival T1 (days)	Arrival T2 (days)	Δ*t*_1_ (hr)	Δ*t*_2_ (hr)	V_1_ (m/s)	V_2_ (m/s)
1	15.5677	16.2771	9.378	26.405	314.8	309.3
2	15.5678	16.2765	9.381	26.390	314.5	309.5
3	15.6007	16.25	10.171	25.754	309.0	309.7
4	15.5	16.3576	7.754	28.337	305.7	308.7
5	15.5694	16.2778	9.420	26.421	313.4	309.1
6	15.6493	16.191	11.337	24.338	310.8	312.1
7	15.5104	16.3403	8.004	27.921	312.5	308.7
8	15.5243	16.334	8.337	27.770	307.5	308.1
9	15.5729	16.2889	9.504	26.688	304.4	308.2
10	15.6424	16.2035	11.172	24.638	311.6	310.0
11	15.6354	16.2188	11.004	25.005	306.8	309.7
12	15.6389	16.2083	11.088	24.753	310.7	310.0
13	15.6146	16.2396	10.505	25.505	308.2	309.0
14	15.566	16.3056	9.338	27.089	306.7	304.8
15	15.4965	16.3611	7.670	28.421	309.5	307.7
16	15.6007	16.2361	10.171	25.421	316.4	310.9
17	15.6493	16.2014	11.337	24.588	308.4	310.0
18	15.4757	16.3958	7.171	29.253	312.0	303.6
19	15.5278	16.3333	8.421	27.753	309.2	306.8
20	15.5729	16.2986	9.504	26.921	303.6	305.9
21	15.5785	16.2639	9.638	26.088	314.4	310.1
22	15.5694	16.2743	9.420	26.337	315.4	309.4
23	15.5451	16.3056	8.837	27.089	312.1	308.7
24	15.5556	16.2917	9.089	26.755	312.5	309.5
25	15.3681	16.4965	4.589	31.670	304.4	307.0
26	15.5313	16.3306	8.505	27.689	304.1	308.2
27	15.6007	16.2396	10.171	25.505	313.2	311.1
28	15.5451	16.3056	8.837	27.089	312.0	308.7
29	15.5451	16.3056	8.837	27.089	310.1	309.3
30	15.5764	16.2639	9.588	26.088	315.9	310.1
31	15.5382	16.3264	8.671	27.588	302.6	308.0
32	15.6319	16.2153	10.920	24.921	311.5	309.7
33	15.6285	16.2257	10.838	25.171	305.1	310.4
34	15.5139	16.3507	8.0878	28.171	303.0	307.7
35	15.5486	16.3056	8.921	27.089	306.0	309.7
36	15.559	16.3021	9.170	27.005	304.5	308.4
37	15.5938	16.25	10.005	25.754	306.2	312.8
38	15.6424	16.2118	11.172	24.837	304.4	310.8
39	15.6632	16.184	11.671	24.170	308.0	311.3
40	15.6458	16.1944	11.253	24.420	314.1	310.6

The arrival time of the second signal at Site 1 was 06:39:00 UTC on 16 January (or 16.2771 days in Jan., Table 5) and about 26.405 h after the major eruption. The distance traveled by the wave through Route 2 (Figure 1B) is approximately 29,403 km and the estimated propagation speed is estimated to be 309.3 m/s (Table 5), a value consistent with the speed of the first signal, although slightly smaller.

### Shockwave speed estimate at 189 weather stations

The 1-min interval data (from 151 stations) are examined to identify the possible shockwave signals. We use the peak signal to determine the time of signal for the wave propagation speed computation. The 5-min interval data from the rest 38 stations have much lower resolution than the Sites 1 and 2 data and the 1-min interval data. For these 5-min interval data, we first smooth the time series using a 4-point digital finite impulse response (FIR) filter (Proakis and Manolakis, 1992), which allows a better resolution of the signals from the shockwaves.

**Figure 4:**
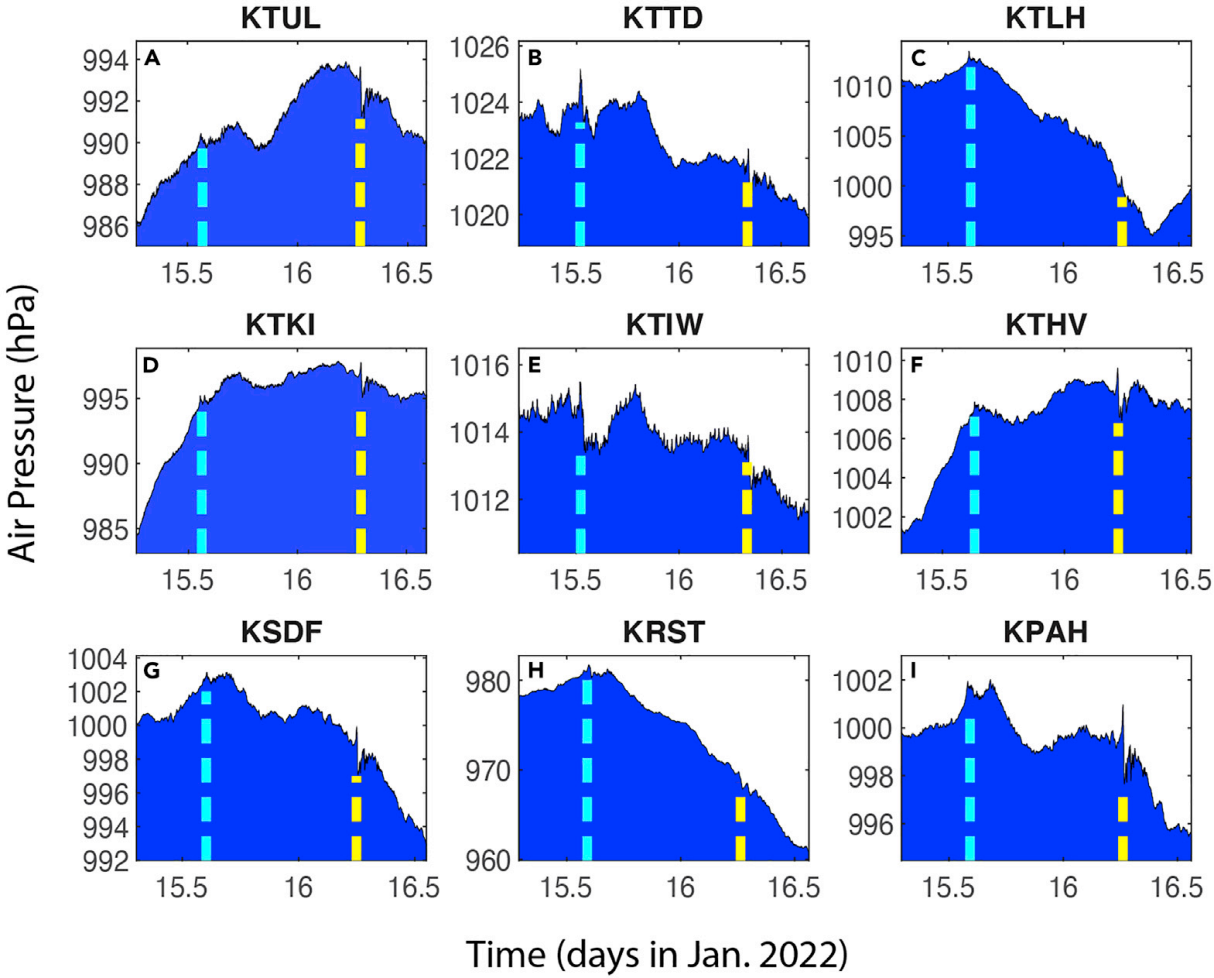
Examples of the 1-min data showing the shockwave signals (I) Examples of 1-min interval air pressure times series at 9 of the 191 stations (Table 1). The vertical bars indicate the timings of the first (red) and second (black) arrivals of the shockwave signals using the estimated propagation speed of 309.5 m/s from Sites 1 and 2 (Table 1). Panels A–I are for different stations.

**Figure 5:**
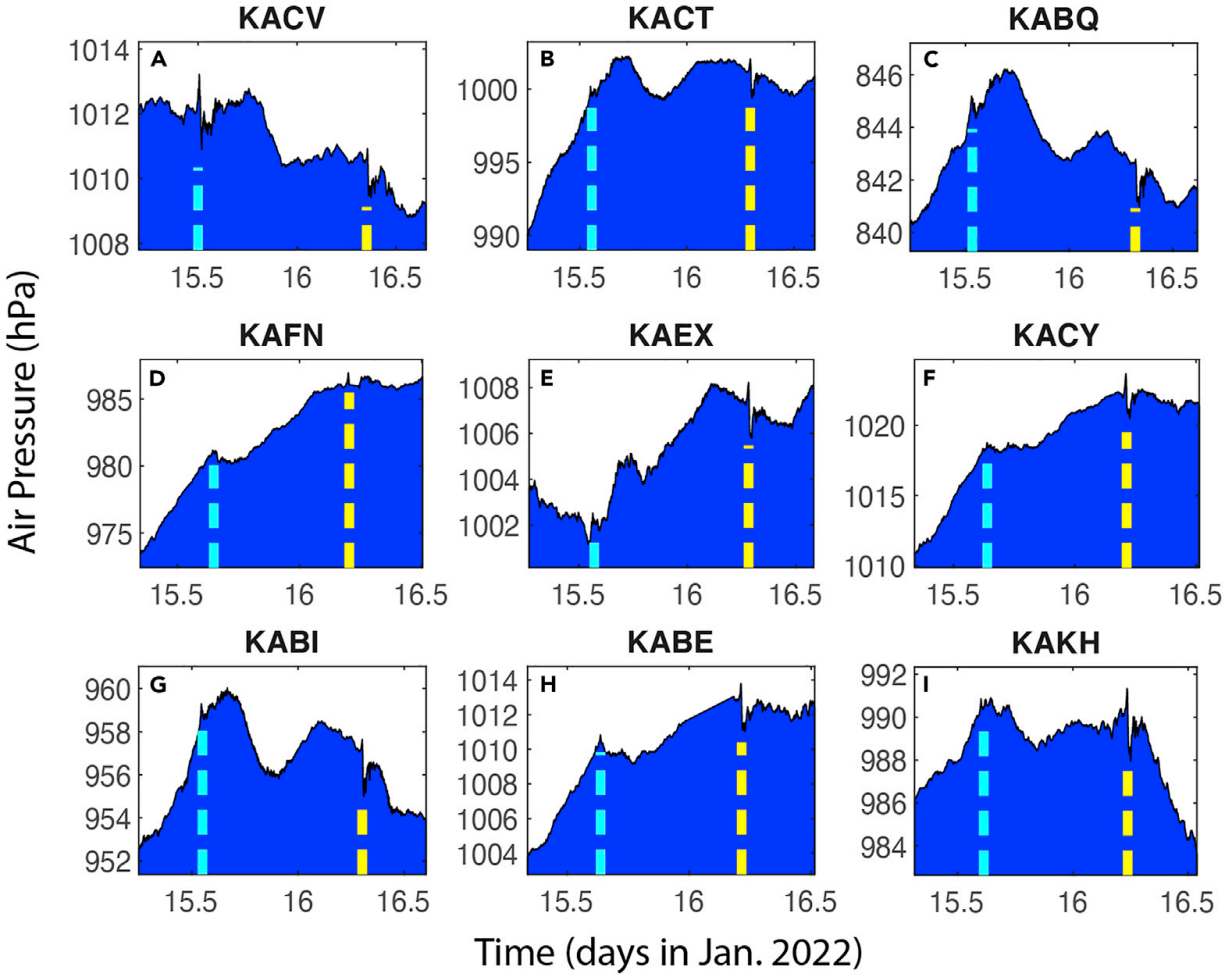
Examples of the 1-min data showing the shockwave signals (II) Same as Figure 4 for additional stations. Panels A–I are for different stations.

**Figure 6:**
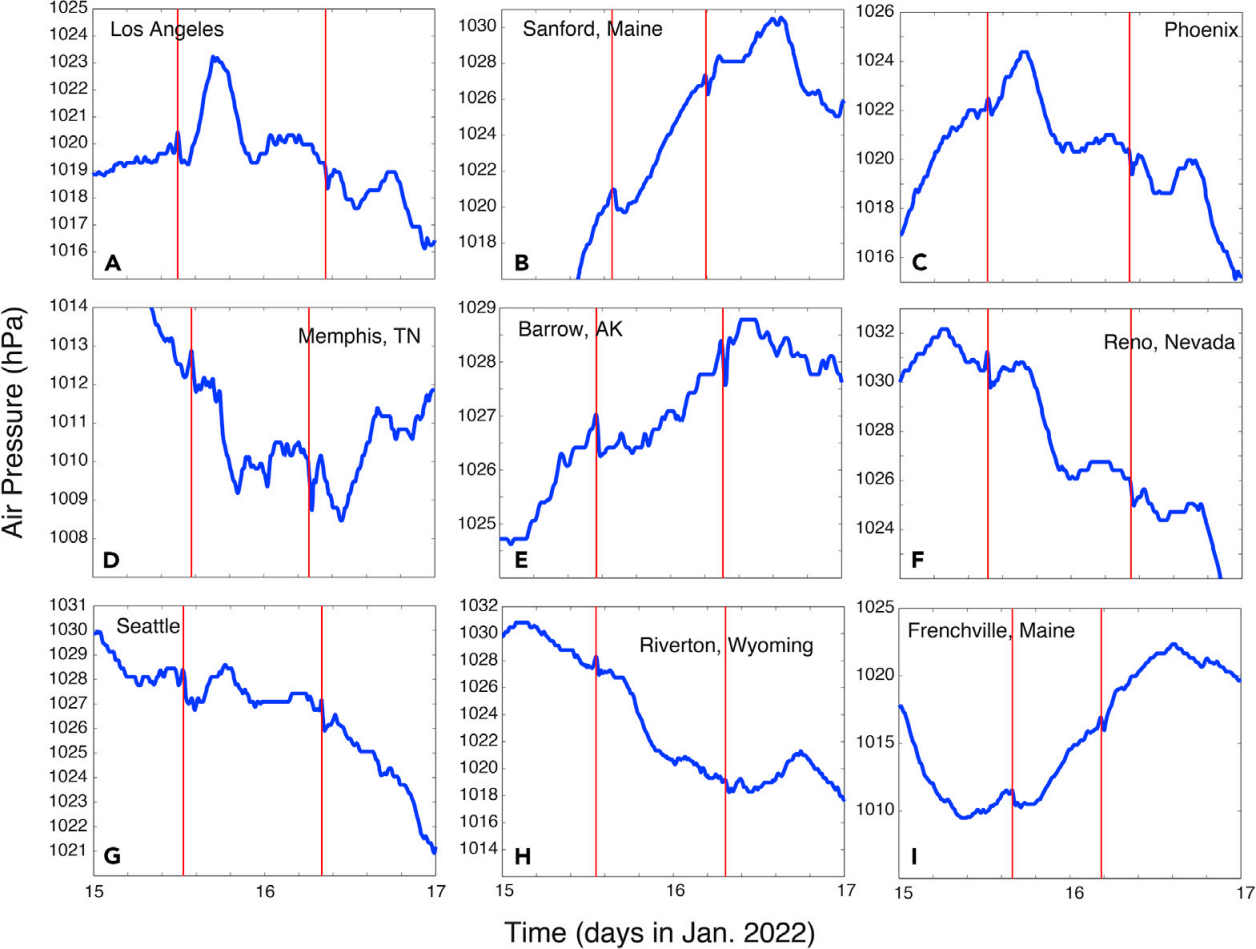
Examples of the 5-min data showing the shockwave signals The 5-min interval air pressure times series at 9 stations (Table 1). The raw data were treated by a 4-point or 15 min moving average filter twice (back and forth, to eliminate the phase shift). The red bars indicate the timings of the first and second arrivals of the shockwave signals. Panels A–I are for different stations.

**Figure 7:**
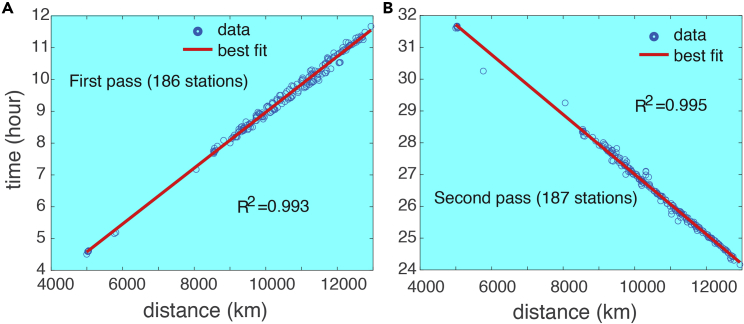
Shockwave speed computation Regression of the spherical distance of the stations to the volcano and the arrival times of the first signal (A) and second signal (B).

The time series data from most of the 189 stations show at least two signals (Tables 5, 6, 7, and 8) that are consistent with the arrival of the first and second shockwave signals from the eruption through Routes 1 and 2 (Figure 1B), respectively. Figures 4 and 5 show some examples from the 1-min data, while Figure 6 shows some examples from the smoothed 5-min data. Based on the timings of these signals, and the locations of the stations, we computed the propagation speed for both signals at each station (Tables 5, 6, 7, and 8). The method is consistent for all stations: the peak time is used for the computation of the signal arrival time. For all stations, the average speed for the first signal is 309.7 ± 4.2 m/s, and that for the second signal is 309.2 ± 1.5 m/s. The second signals are consistently greater in amplitude. Many of the stations show negative changes in the second signal. The standard deviation (1.5 m/s) for the second signal of the computed speed is only about ⅓ of that of the first signal (4.2 m/s). These velocity values are consistent with the sound wave speed in the upper trosphere and stratosphere (more discussion later). These results demonstrate that it is almost certain that these signals are from eruption-induced shockwaves propagating around the globe. The vertical lines in Figures 4, 5, and 6 are the arrival times of the first two signals using the averaged propagation speed of 309.5 m/s (Table 8). Figure 7 shows the regressions of the computed arrival time and distance from the eruption site. The R^2^ values are as high as 0.993 and 0.995 for the first and second signals, respectively.

**Table 6:** Arrival time of shockwaves, time of travel, and propagation speed. For stations with data sampled at 1-min intervals, Part I

Station No.	Arrival T1 (days)	Arrival T2 (days)	Δ*t*_1_ (hr)	Δ*t*_2_ (hr)	V_1_ (m/s)	V_2_ (m/s)
41	15.651	16.193	11.371	24.389	309.5	311.6
42	15.656	16.190	11.489	24.305	308.8	311.5
43	15.365	16.494	4.505	31.603	308.0	307.9
44	15.369	16.494	4.620	31.603	303.6	307.5
45	15.368	16.496	4.589	31.653	304.3	307.2
46	15.369	16.494	4.603	31.603	304.6	307.5
47	15.369	16.494	4.603	31.620	304.5	307.3
48	15.368	16.497	4.589	31.670	305.1	306.9
49	15.368	16.496	4.589	31.653	303.9	307.2
50	15.392	16.438	5.155	30.254	310.8	314.6
51	15.393	–	5.189	–	311.1	–
52	15.526	16.337	8.388	27.837	307.1	306.9
53	15.534	16.331	8.570	27.689	307.3	306.5
54	15.518	16.342	8.189	27.955	310.5	306.8
55	15.530	16.329	8.472	27.655	308.2	307.7
56	15.568	16.301	9.389	26.988	305.0	305.9
57	15.540	16.317	8.705	27.355	310.7	307.6
58	15.528	16.336	8.421	27.821	313.3	304.9
59	15.543	16.322	8.789	27.470	307.5	306.4
60	15.530	16.334	8.472	27.770	307.1	306.7
61	15.526	16.333	8.388	27.753	310.5	306.8
62	15.527	16.320	8.405	27.437	309.3	310.5
63	15.527	16.319	8.405	27.420	310.5	310.4
64	15.535	16.324	8.604	27.537	308.1	307.5
65	15.521	16.344	8.253	28.005	307.0	306.6
66	15.519	16.340	8.205	27.921	310.7	306.9
67	15.537	16.324	8.637	27.521	308.3	307.3
68	15.551	16.319	8.971	27.403	302.8	306.6
69	15.568	16.306	9.389	27.105	305.1	304.6
70	15.567	16.286	9.372	26.621	308.2	309.2
71	15.581	16.269	9.705	26.205	311.1	309.1
72	15.638	16.211	11.054	24.821	309.9	310.0
73	15.554	16.292	9.055	26.755	314.2	309.3
74	15.594	16.246	10.020	25.653	317.3	309.5
75	15.557	16.289	9.120	26.688	314.6	309.2
76	15.570	16.274	9.437	26.337	315.3	309.2
77	15.562	16.284	9.237	26.570	314.3	309.2
78	15.518	16.339	8.189	27.888	308.2	308.2
79	15.590	16.251	9.921	25.788	314.7	310.1
80	15.554	16.291	9.055	26.738	314.8	309.3
81	15.518	16.335	8.189	27.804	312.1	308.0
82	15.632	16.218	10.920	24.989	309.3	309.8
83	15.605	16.250	10.272	25.754	307.5	309.1
84	15.596	16.254	10.053	25.855	304.8	311.6
85	15.583	16.261	9.737	26.021	316.1	309.1
86	15.646	16.199	11.253	24.537	311.4	310.4
87	15.531	16.331	8.505	27.689	304.1	308.2
88	15.615	16.221	10.521	25.053	318.0	310.3
89	15.550	16.296	8.954	26.853	313.9	309.4
90	15.616	16.219	10.538	25.020	317.9	310.5
91	15.564	16.296	9.288	26.853	304.7	308.7

**Table 7:** Arrival time of shockwaves, time of travel, and propagation speed. For stations with data sampled at 1-min intervals, Part II

Station No.	Arrival T1 (days)	Arrival T2 (days)	Δ*t*_1_ (hr)	Δ*t*_2_ (hr)	V_1_ (m/s)	V_2_ (m/s)
92	15.604	16.240	10.255	25.505	310.7	311.1
93	15.608	16.230	10.337	25.272	317.7	310.1
94	15.615	16.237	10.505	25.437	308.5	309.7
95	15.549	16.310	8.921	27.204	306.5	308.2
96	15.633	16.216	10.953	24.938	309.7	309.8
97	15.499	16.359	7.737	28.370	307.2	308.2
98	15.626	16.224	10.788	25.121	309.1	309.9
99	15.557	16.299	9.120	26.937	307.0	308.9
100	15.622	16.229	10.687	25.238	309.0	309.7
101	15.601	16.253	10.188	25.821	307.6	309.3
102	15.498	16.358	7.704	28.353	308.3	308.4
103	15.552	16.294	9.005	26.803	314.0	309.4
104	15.578	16.280	9.621	26.472	305.6	309.0
105	15.630	16.219	10.872	25.003	309.5	310.1
106	15.554	16.294	9.055	26.803	313.4	309.0
107	15.630	16.218	10.872	24.989	310.4	310.0
108	15.535	16.322	8.587	27.470	308.9	308.2
109	15.649	16.196	11.321	24.453	311.7	310.4
110	15.592	16.245	9.972	25.637	320.0	309.3
111	15.602	16.237	10.205	25.437	317.2	309.9
112	15.548	16.308	8.904	27.153	308.7	308.3
113	15.592	16.264	9.972	26.088	306.9	308.9
114	15.531	16.317	8.505	27.372	312.6	309.1
115	15.619	16.233	10.603	25.337	308.8	309.7
116	15.605	16.233	10.272	25.353	316.6	310.3
117	15.590	16.251	9.921	25.788	316.8	309.3
118	15.563	16.288	9.254	26.654	306.1	310.9
119	15.546	16.302	8.853	27.005	305.9	311.5
120	15.549	16.311	8.937	27.221	304.6	308.5
121	15.558	16.303	9.137	27.021	304.6	308.5
122	15.589	16.254	9.888	25.838	316.7	309.1
123	15.499	16.358	7.721	28.353	308.2	308.2
124	15.644	16.204	11.220	24.638	310.5	309.9
125	15.547	16.315	8.870	27.305	304.5	308.3
126	15.572	16.274	9.470	26.321	315.1	309.1
127	15.532	16.330	8.520	27.672	304.2	308.2
128	15.525	16.332	8.354	27.720	309.7	307.8
129	15.620	16.235	10.637	25.404	306.3	309.5
130	15.617	16.233	10.572	25.353	309.2	309.6
131	15.575	16.280	9.554	26.472	306.9	309.3
132	15.584	16.257	9.770	25.920	316.5	309.7
133	15.501	16.352	7.771	28.205	307.3	309.6
134	–	16.262	–	26.037	–	309.0
135	15.529	16.333	8.438	27.737	304.5	308.3
136	15.606	16.246	10.303	25.653	304.2	311.3
137	15.585	16.267	9.804	26.172	303.9	311.0
138	15.591	16.249	9.938	25.721	316.9	309.9
139	15.605	16.254	10.272	25.838	304.0	309.5
140	15.516	–	8.138	–	308.7	–
141	15.544	16.313	8.820	27.271	306.4	308.6

**Table 8:** Arrival time of shockwaves, time of travel, and propagation speed. For stations with data sampled at 1-min intervals, Part III

Station No.	Arrival T1 (days)	Arrival T2 (days)	Δ*t*_1_ (hr)	Δ*t*_2_ (hr)	V_1_ (m/s)	V_2_ (m/s)
142	15.572	16.273	9.487	26.304	314.5	309.3
143	15.642	16.213	11.155	24.854	306.5	309.8
144	15.600	16.254	10.154	25.838	308.2	309.2
145	15.613	16.238	10.471	25.454	309.2	309.7
146	15.648	16.203	11.304	24.621	307.6	310.4
147	15.622	16.229	10.670	25.238	305.8	311.3
148	15.599	–	10.121	–	311.3	–
149	15.504	16.353	7.838	28.221	303.5	309.7
150	15.550	16.306	8.954	27.103	307.1	308.8
151	15.628	16.223	10.821	25.104	309.3	309.6
152	15.612	16.240	10.437	25.521	308.4	309.6
153	15.622	16.231	10.687	25.305	304.7	310.7
154	15.597	16.241	10.087	25.538	308.7	313.5
155	15.587	16.254	9.837	25.855	316.6	309.6
156	15.588	16.268	9.854	26.189	306.4	309.3
157	15.597	16.243	10.070	25.589	315.7	310.3
158	15.542	16.304	8.755	27.038	315.0	309.3
159	15.529	16.328	8.455	27.621	307.9	308.3
160	15.540	16.314	8.705	27.288	309.8	308.7
161	15.592	16.260	9.955	26.004	306.6	310.2
162	15.544	16.307	8.820	27.120	311.6	308.7
163	15.641	16.206	11.138	24.689	310.9	310.1
164	15.620	16.233	10.637	25.337	308.3	309.4
165	15.616	16.220	10.538	25.037	318.5	310.1
166	15.558	16.300	9.153	26.954	305.1	308.9
167	15.602	16.235	10.205	25.404	317.8	310.0
168	15.562	16.290	9.237	26.705	306.1	310.5
169	15.594	16.244	10.020	25.620	317.3	309.9
170	–	16.240	–	25.521	–	310.0
171	15.624	16.228	10.721	25.221	308.8	309.6
172	–	16.294	–	26.803	–	309.2
173	15.645	16.199	11.237	24.537	311.8	310.4
174	15.565	16.281	9.305	26.489	314.5	309.3
175	15.638	16.210	11.071	24.804	309.7	310.1
176	15.506	16.356	7.905	28.289	303.8	308.2
177	15.551	16.296	8.971	26.853	313.9	309.2
178	–	16.194	–	24.420	–	310.3
179	15.591	16.250	9.938	25.754	315.6	310.0
180	15.581	–	9.689	–	305.6	–
181	15.529	16.320	8.455	27.437	312.9	308.9
182	15.544	16.302	8.820	27.005	313.8	309.3
183	15.636	16.213	11.021	24.854	309.9	310.0
184	–	16.346	–	28.053	–	308.2
185	15.566	16.288	9.338	26.654	309.6	308.7
186	15.588	16.255	9.871	25.872	313.9	310.0
187	15.584	16.258	9.770	25.953	315.2	309.8
188	15.639	16.210	11.088	24.804	309.6	309.9
189	15.585	16.274	9.787	26.321	305.7	308.8
190	15.531	16.313	8.503	27.271	314.7	309.6
191	15.586	16.255	9.821	25.872	315.1	310.2
			Average		309.7	309.2
			Standard deviation		4.2	1.5
			Overall average		309.5	
			Standard deviation		2.9

### A global view of the arrival time

**Figure 8:**
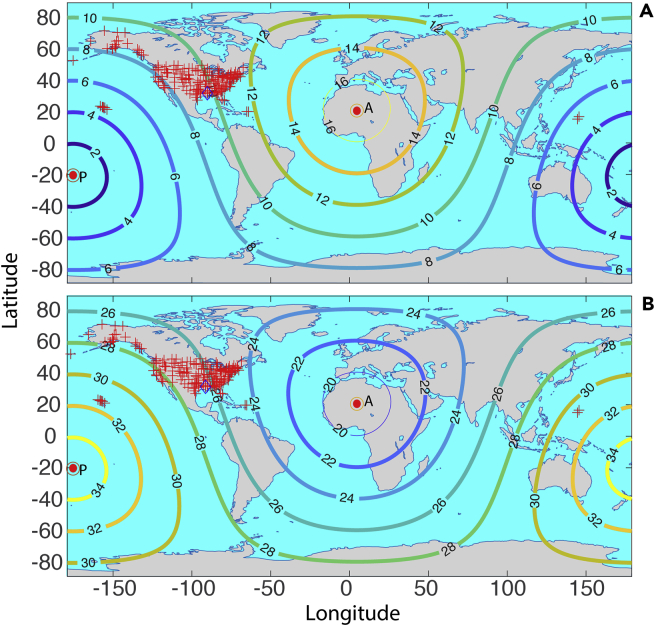
The global distribution of arrival time of shockwaves from the regression model The arrival time (hours) distribution using the average propagation speed from the computation on the globe for (A) the first signal and (B) the second signal through Routes 1 and 2, respectively. Points P and A are the location of the volcano and its antipole, which is a point inside southern Algeria, Africa. The red crosses are the locations of weather stations. The diamond shows the locations of the two stations with high-resolution data in Louisiana, U.S.

**Figure 9:**
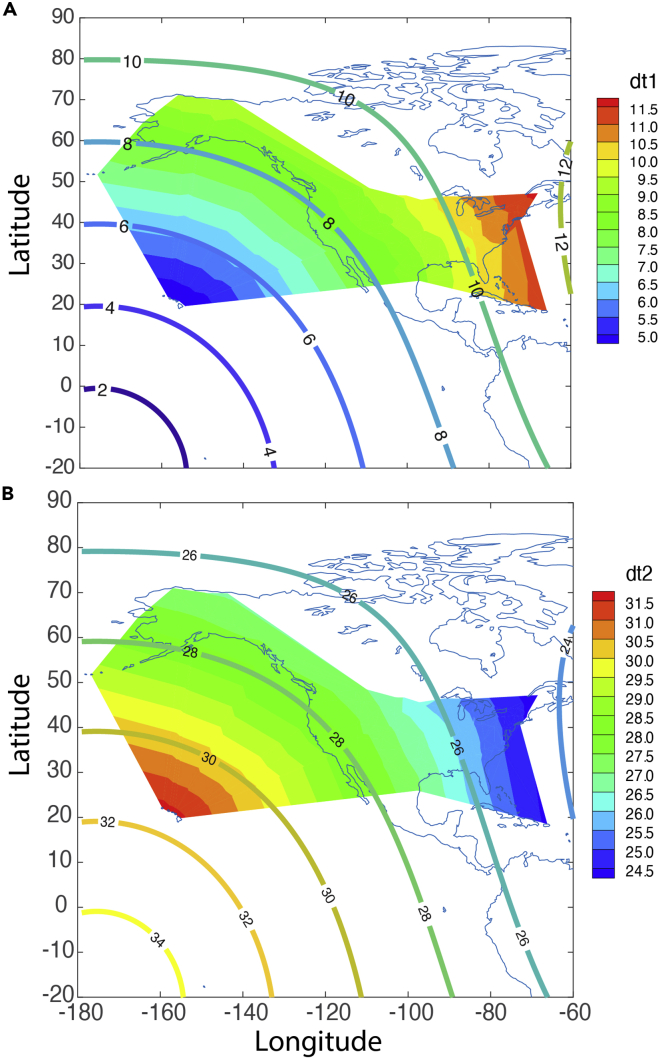
The observed arrival times of shockwave signals compared with those from the regression Arrival time (hour) for (A) the first and (B) the second signal measured at the weather stations (the colored area) through Routes 1 and 2, respectively. The line contours are the theoretical SWs using the averaged velocity of Tables 5, 6, 7, and 8. Note that the colors of the lines are not corresponding to the color bar scales; only the labeled values are relevant.

Using the average propagation speed of 309.5 m/s for the first and second passes of the shockwaves, the global distribution of arrival times is computed assuming a spherically propagating wave for the first (Figure 8A) and second (Figure 8B) signals. The observed arrival time (Figure 9A) of the first signal among the 191 stations roughly ranges between 4.5 and 11.7 h after the eruption; while that for the second signal (Figure 9B) ranges roughly between 24.3 and 31.7 h after the eruption. All these show consistent results that the recorded fluctuations of air pressure time series data at the 191 sites are from the eruption of the Hunga Tonga-Hunga Ha’apai underwater volcano.

### Signals for the 3^rd^ – 6^th^ passes

**Figure 10:**
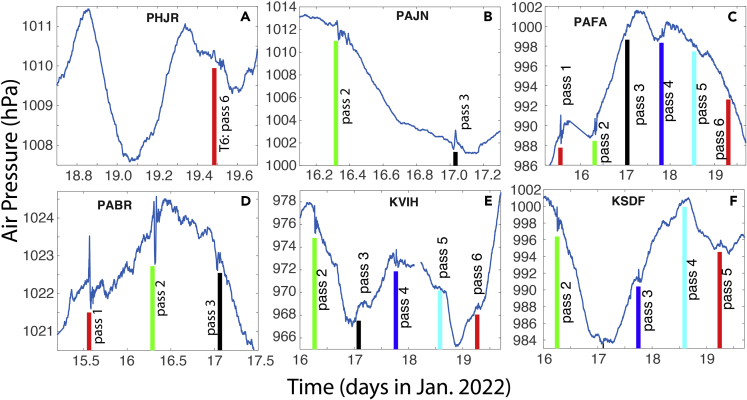
Recorded signals showing multiple passes of the shockwaves Multiple passes of the shockwaves: (A–F) are for six different stations. The vertical bars indicate the timings of the anticipated shockwave arrivals (2^nd^ to 6^th^ signals as marked) using the average speed we computed (309.5 m/s, Table 8).

Although data recorded at Sites 1 and 2 with the shortest ensemble sampling intervals do not show any sign of the signal after the second pass, many stations did show signals for the 3^rd^ through the 6^th^ passes. This is clear from the 1-min data but not the 5-min data. Figure 10 shows some examples of the time series with the anticipated timings of the shockwaves and the numbers of passes indicated by the vertical lines. As we can see that there are great variabilities in the amplitude among stations but there is one thing in common, that is the signals of the shockwaves at many stations appeared multiple times as the waves traveled around the globe. The timings are quite good approximations using the averaged speed (309.5 m/s, Table 8).

## Discussion

### Reliability of the signals

From the analysis, the air pressure data from these 191 stations show double and/or multiple signals that are remarkably consistent with spherical shockwave propagation on a global scale. Is it possible that all these were coincidental? Assume that there is an α probability for a signal to randomly match the timing of the arrival of the first or second signal at a time consistent with the arrival of a shockwave propagating around the globe. To have both signals matching the times of the two passes of the shockwaves, it would have a probability of $\alpha^{2}$. For all the n stations to match them by chance, the probability is

(Equation 1) $$p_{n}={\left(\alpha^{2}\right)}^{n}$$

In our case, there are a few stations that did not record data showing both signals (for the first and second pass) due to data gaps. Considering that, n=186 (instead of 191), the probability of match is $p_{186}=\alpha^{372}$. Even if there was a high probability of 0.5 chance of one match, having all 186 stations match by chance for both signals for this event would have a negligible probability of $∼10^{-112}$. This estimate does not even consider the fact that over 70% of the stations with 1-min data show signals from the 3^rd^, 4^th^, 5^th^, or even 6^th^ passes. Of course, the real probability of one match can be reasonably assumed to be much smaller than 0.5, and the combined event being a coincidence is essentially impossible.

### Propagation speed of shockwaves in the atmosphere

Infrasound and shockwaves are acoustic or pressure waves propagating at the speed of sound in the atmosphere (Fee and Matoza, 2013). The speed of sound in an idealized gas (Salomons, 2001) is:

(Equation 2) $$c=\sqrt{\frac{\partial p}{\partial \rho }}=\sqrt{\gamma RT}$$

where ρ is the air density, p is the air pressure, γ∼1.4 is the specific heat ratio, R=287 J/kgK is the universal gas constant, and T is the air temperature in Kelvin. Because the estimated shockwave propagation speed is ∼309 m/s, this corresponds to the sound speed in the upper troposphere where the air temperature is approximately −35°C and −40°C. The air temperature profile is not a constant but variable as a function of time, location, height, season, and in general, atmospheric dynamics. Although the actual dynamical processes and propagation of the shockwaves need to be illustrated by a proper mathematical model, it is well known that the vertical profile of sound speed in the atmosphere has a minimum around the top of the troposphere and lower stratosphere (Piece, 1989; Fee and Matoza, 2013). This provides an idealized environment for a waveguide or sound channel within which sound can propagate to a far distance without major dissipation.

### Ringing after the shockwaves

**Figure 11:**
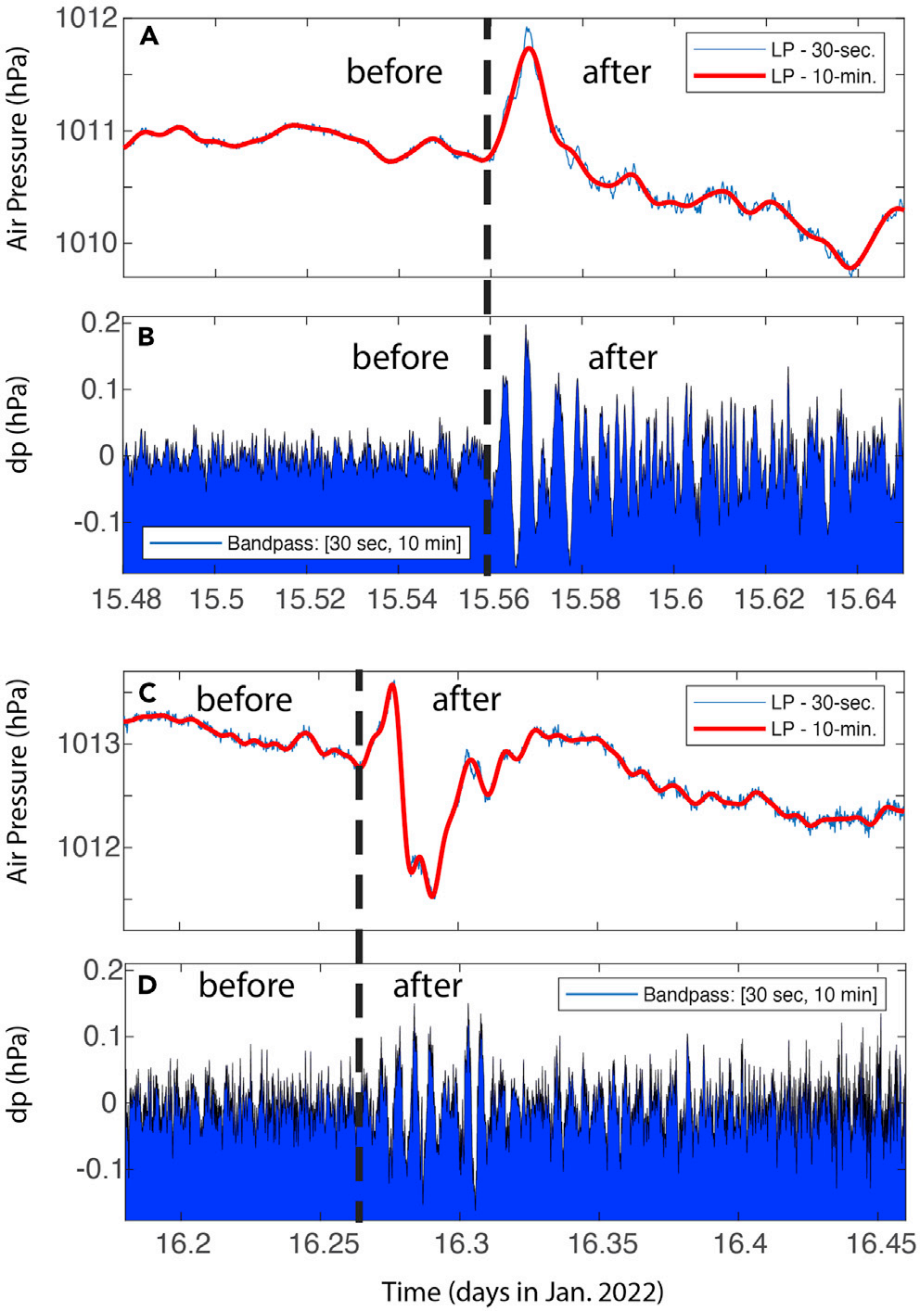
The post-arrival ringing of signals at Site 1 (A) the first signal - before and after its arrival; the blue and red lines are low-pass filtered versions of the raw data with 30-s and 10-min cutoff periods, respectively; (B) is the band-pass filtered results using the Fourier filter (O’Haver, 2022) for periods between 30 s and 10 min, showing the ringing after the arrival of the wave; (C) and (D) are similar, except that they correspond to the second signal period. Data from Site 2 are similar (figures omitted).

In the air pressure time series data from Sites 1 and 2, it is apparent that higher-frequency ringing (oscillations) occurred immediately after the shockwave signals (Figure 3). Previous studies on great explosions also indicated ringing after the arrival of the initial disturbance (e.g., Whipple, 1930, as one of the earliest examples). To examine this ringing, we performed an additional analysis of the filtered data and spectrum of the data before and after the main shockwaves. To better visualize the ringing, a band-pass filter is used on the original time series data (Figure 11): it is a Fourier filter (O’Haver, 2022) with cutoff periods of 30 s and 10 min.

**Figure 12:**
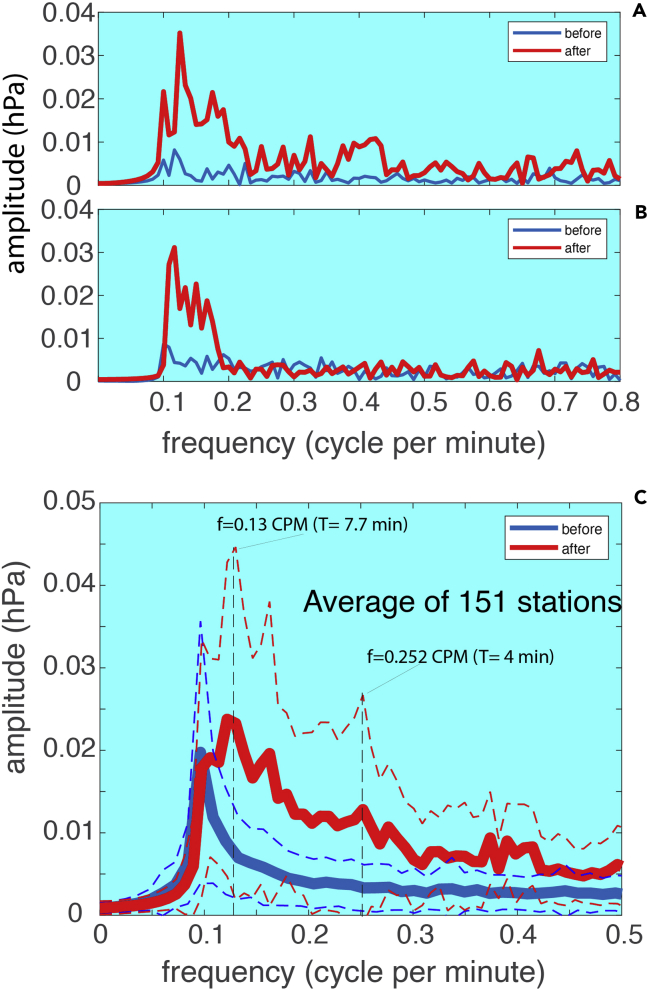
Spectrum comparison to show the shockwave ringing The blue lines are the spectra before the arrival of the shockwaves while the red lines are the spectra after the arrival of the shockwaves: (A) for the first signal for Site 1 (Table 1); (B) for the second signal for Site 1; (C) averaged spectrum for all 151 stations with 1-min interval data. The dashed blue lines show 1 standard deviation above and below the mean before the arrival of the first shockwaves; while the dashed red lines show 1 standard deviation above and below the mean after the arrival of the shockwaves. Results for Site 2 are similar.

Using the arrival time of the signal, the time series was divided into two parts: one *before* and one *after* the arrival of the signal (Figure 11). Indeed, it is obvious that after the arrival of the shockwaves, identifiable oscillations appear to be greater than the background noise before the event (Figures 11B and 11D). For convenience, we converted the Fourier transform coefficients to Fourier series coefficients so that the unit is the same as the data (hPa, Figure 12). The spectrum of the air pressure before the shockwave's arrival (blue lines in Figure 12) has a much lower magnitude than those after (red lines in Figure 12). This is true for both signals, confirming that the ringing of the shockwaves has increased energy. The ringing after the first signal appears to have a broader spectrum (Figure 12A) than that after the second (Figure 12B), indicating the dissipation of high-frequency oscillations with a longer distance for the second signal.

The ringing in the first signal has peak frequencies at ∼ 0.125, 0.175, and 0.217 cycles per minute. These frequencies correspond to periods of 8, 5.7, and 4.6 min, respectively. The ringing in the second signal has a narrower band with low frequencies and lower magnitude for all frequencies. The first few major frequency peaks are at ∼ 0.12, 0.13, 0.15, and 0.17 cycle per minute. These frequencies correspond to periods 5.9 to 8.3 min. The overall spectra however are continuous. The results are consistent with those from Britain and Ireland where oscillations at 6–8 min intervals were reported by Burt (2022).

The 5-min interval data are too sparse to show the ringing. The 1-min interval data are sufficient to resolve the ringing. Among the 151 stations that provided the 1-min interval data, about 110 (∼73%) of them showed clear ringing and significant contrast in oscillation energy between the data before and after the arrival of the shockwaves. The rest 27% do not show obvious differences. To gain a general view of this, we have computed the spectra for the bandpass filtered time series data for all the 151 stations for the first pass of the shockwaves with a 2-h length before and after the shockwaves, respectively. The bandpass filter is the same as the one applied to the high-resolution data from Sites 1 and 2. The averaged spectra are shown in Figure 12C, together with 1 standard deviation below and above the mean, respectively. It is confirmed that the air pressure after the arrival of the shockwaves had significantly higher (>100%) energy oscillations compared to that before the shockwaves, for the majority of the stations. This is particularly true for periods between 4 and 8 min. The spectra, however, are continuous and the oscillations are not simple sinusoidal variations with discrete frequencies. The spectra comparisons for the second pass of the shockwaves and those for Site 2 are similar and the figures are omitted here.

### Spherical waves

**Figure 13:**
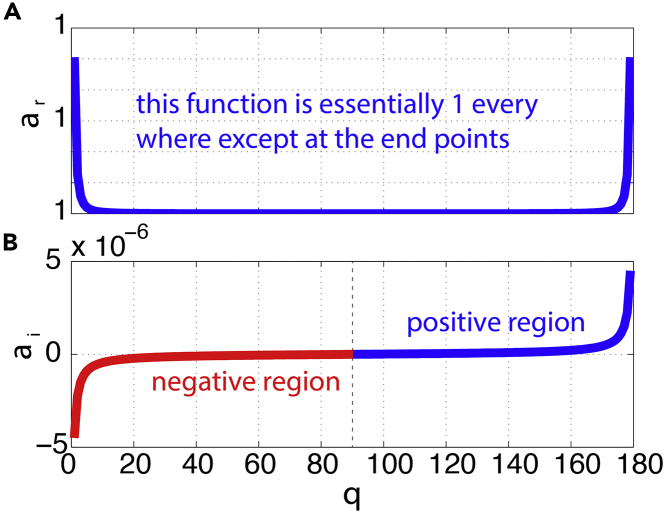
Spherical wave dispersion relation The real and imaginary parts of the factor α in the dispersion relationship: (A) is the real part, which is essentially 1, except when it is at the singular points 0 or 180°; and (B) is the imaginary part as functions of the azimuthal angle θ.

Spherical waves (different from the cylindrical Lamb waves, Pekeris, 1939) are those propagating on the spherical surface of the Earth in the atmosphere. The satellite images (Bachmeier, 2022) are perhaps the first visual evidence and direct observations of the catastrophic volcano eruption-induced shockwaves being remarkable spherical waves. The numerical model simulations (Amores et al., 2022) provide additional support through the dynamics framework that the shockwaves are essentially spherical in nature. The early study of the Krakatoa eruption-induced shockwaves already implied that the waves traveled around the world multiple times must have been spherical waves (Symons, 1888; Gabrielson, 2010), although there was no direct visual evidence. Observations of the Tonga eruption induced shockwaves reverberating around the Earth multiple times (e.g., Burt, 2022 and resulted presented here) are consistent with the satellite images. This means that the shockwaves, after being generated by the enormous explosion, must be bending their rays on the Earth’s surface and travel in a spherical form (rather than expanding in a cylindrical way like the Lamb wave). This can be verified by considering a linear sound wave model in the atmosphere. The small aspect ratio of the atmosphere (thin layer) and the small curvature (relatively large radius of the Earth) make the wave spherical on the Earth's "surface" (in the atmosphere). The method section derives the wave propagation dispersion relationship which indicates that (1) the propagation speed or celerity U is not exactly the same as the speed of sound *c* because of the curvature of the Earth (or the radius of the Earth *r* being finite, not infinity); (2) the waves are non-dispersive—meaning that the wave propagation speed is not dependent on the frequency which allows the waves to travel long distance without major dissipation, as the multiple passes of the waves demonstrated. We can see from the method section (Equation 9) that the celerity of the spherical wave is the speed of sound multiplied by a factor α:

(Equation 3) $$\alpha =\sqrt{1-i\frac{1}{r}\cot\;\theta }=\alpha_{r}+i\alpha_{i}$$

where $\alpha_{r}$ is the real part of α and $\alpha_{i}$ is the imaginary part of α. The actual spherical wave propagation speed is $\alpha_{r}$ multiplied by c while $\alpha_{i}$ multiplied by c gives the exponent for the change in the amplitude of the wave. To provide an intuition for this, we plotted the functions (Figure 13). At 1° and 179° polar angles, the real part is 1.000000000010108, a value very close to 1 (Figure 13A). Thus, it can be seen that the real part is essentially 1 except at two singular points: the pole and antipole (Figure 13A), which means that *the shockwave propagation speed is essentially the speed of the sound*. The imaginary part has the physical meaning of the exponent, which influences the amplitude of the wave. The factor is

(Equation 4) $$f=e^{i\left(r\theta -c\alpha_{r}t\right)}e^{c\alpha_{i}t}∼e^{i\left(r\theta -ct\right)}e^{c\alpha_{i}t}$$

The average radius of the Earth *r* = 6,371,000 m, and the second term in the square root of Equation 3 is much smaller than 1 (except at the singular points of 0 and 180°):

(Equation 5) $$\frac{1}{r}\cot\theta \ll 1,\;\left(\theta \neq 0,\;\theta \neq 180{}^{\circ}\right)$$

Using Taylor series expansion, Equation 3 gives:

(Equation 6) $$\alpha \approx 1-i\frac{1}{2r}\cot\;\theta$$

This gives an expression of the imaginary part

(Equation 7) $$\alpha_{i}\approx -\frac{1}{2r}\cot\;\theta$$

The imaginary part $\alpha_{i}$ is negative when the azimuthal angle is less than 90° but positive when it is greater. This is consistent with the computation using (Equation 3), as shown in Figure 13B. This is expected because when the azimuthal angle is less than 90°, the radius of the wave increases and the amplitude of the wave should decrease even without dissipation, whereas after passing the 90° azimuthal angle, the radius starts to decrease thereby causing the amplitude to increase again under an idealized situation (no dissipation). Note that the plots in Figure 13 are obtained by specifying the azimuthal angle to be between 1° and 179° to avoid singular points at the pole (0°) and antipole (180°). In mathematics, an impulse signal from a point source is a delta function that is infinite in magnitude at that point, but an integration over an arbitrarily small region encompassing the point gives a finite value (Bracewell, 2000). Even though our analysis shows that the shockwave propagation speed is consistent with the speed of sound, the data do not show a clear amplitude variation (i.e. decreasing before the azimuthal angle reaching 90° and increasing after 90°). This may indicate that the actual mechanism is more complicated than the linear model if the shockwaves propagate in an atmospheric waveguide, given the minimum air temperature at the top of the troposphere and the lower stratosphere. A numerical model can be a useful tool to illustrate the mechanism better.

### Conclusions

The 15 January 2022 eruption of the Hunga Tonga-Hunga Ha’apai underwater volcano generated global-scale spherical shockwaves in the atmosphere recorded by air pressure sensors at meteorological stations. The present study included stations that are more than 5000–12000 km away in the subtropical Pacific (Hawaii and Guam), Alaska, the contiguous U.S., and Puerto Rico. Most of the 191 stations recorded the first two signals with the timing consistent with the arrival of the shockwaves for the first and second passes through Routes 1 and 2. This preliminary analysis allows us to conclude that:

1. The probability of the data randomly having the two peaks at the “right time” for the two signals of shockwaves from the Tonga underwater volcano eruption through the two routes at 191 different stations thousands of kilometers apart is almost impossible. These signals must have been caused by the eruption on January 15, 2022.
2. The estimated shockwave propagation speed is 309.5 ± 2.9 m/s. The small standard deviations and consistency in speed for both signals indicate that the shockwaves propagated radially and symmetrically as spherical (not cylindrical or Lamb) waves at the first-order approximation.
3. More than 70% of the stations with 1-min interval data also recorded multiple passes of the shockwaves, at least for the third to 6^th^ passes.
4. These wave speed values are consistent with the acoustic wave speed in the upper troposphere and lower stratosphere, although our data and analysis are not sufficient to determine more specific dynamics, which would be suitable subjects for study with numerical modeling.
5. Ringing occurred after the arrival of the major wave disturbances. These ringings had periods of 4–8 min that are resolvable by the high-resolution data and the 1-min interval data. A comparison of the spectra before and after the peak signal indicated that the ringings have continuous spectra. The spectrum of the second signal is narrower, possibly due to the longer traveling distance-related dissipation of higher frequency components.
6. The analysis of the dispersion relationship for spherical wave propagation indicates that the spherical shockwaves is non-dispersive and should propagate essentially at the same speed as sound in the air because of the small aspect ratio and Earth’s curvature. This is consistent with the findings of the data analysis.

### Limitation of the study

The dispersion equation of the simple model predicts a change in amplitude: before the azimuthal angle reaches 90°, the amplitude should decrease, and it should increase after 90°. However, this has not been confirmed, which might be because of the noise in the data but also could be a result of complex dynamics not resolvable by the linear model. This may require more high-resolution data to better quantify the magnitude of the signals at different sites, or numerical modeling of the shockwaves.

## Ethics statement

This study does not involve animal or human.

## Funding statement

This research is not funded.

## STAR★Methods

### Key resources table

**Table:** 

REAGENT or RESOURCE	SOURCE	IDENTIFIER
**Deposited data**		

High-resolution air pressure data	This paper.	Li, C. (2022a). High-Resolution Air Pressure Measured from Ground Stations. https://doi.org/10.31390/oceanography_coastal_wavcis.02
Low-resolution air pressure data	NOAA	N/A

**Software and algorithms**		

MATLAB	MathWorks	https://www.mathworks.com
TECPLOT	Tecplot	https://www.tecplot.com
Filtering & spectrum	Li (2022b)	https://doi.org/10.1017/9781108697101
Analysis code	This paper	N/A

### Resource availability

#### Lead contact

Information and requests for resources should be directed to and will be fulfilled by the Lead Contact, Chunyan Li (cli@lsu.edu).

#### Materials availability

N/A.

### Method details

#### Derivation of dispersion relationship of large scale shockwaves in the atmosphere

The linearized acoustic wave equation is written as (Pierce, 1989),

$$\nabla^{2}p-\frac{1}{c^{2}}\frac{\partial^{2}p}{\partial t^{2}}=0$$

where p is the air pressure, and c is the speed of sound, which is determined by

$$c^{2}=\frac{\partial p}{\partial \rho }$$

where ρ is the air density. Since the geostationary satellites showed spherical waves on the Earth’s surface (Bachmeier, 2022; World Bank, 2022), we can use the spherical polar coordinate system to express the Laplacian ($\nabla^{2}$). This leads to (Arfken et al., 2013),

$$\nabla^{2}p=\frac{1}{r^{2}}\frac{\partial }{\partial r}\left(r^{2}\frac{\partial p}{\partial r}\right)+\frac{1}{r^{2}\;\sin\;\theta }\frac{\partial }{\partial \theta }\left(\sin\;\theta \frac{\partial p}{\partial \theta }\right)+\frac{1}{r^{2}{\sin\;}^{2}\;\theta }\frac{\partial^{2}p}{\partial \varphi^{2}}$$

Here, r is the radial distance from the center of Earth to the point of wave disturbance. The variable θ is the polar angle with the location of eruption (P in Figures 1 and 8) as the pole and φ the azimuthal angle. For a problem with global-scale shockwave propagation, the aspect ratio of the motion is small, that is, the thickness of the atmosphere within which the waves propagate is much smaller than the lateral scale over which the wave can propagate on the Earth’s surface. If the waves can reach a height of 20–40 km, half of the perimeter of the Earth is about 20 thousand km, so the aspect ratio is ∼1/500–1/1000. With this small aspect ratio, the waves propagate in a thin layer on the surface of Earth, and the change in r is negligible at the first-order approximation. Likewise, the waves expand radially and symmetrically outward with the location of the eruption as the pole (or center); thus, the dependence on the azimuthal angle can be neglected at the first-order approximation. Based on these assumptions, we have

$$\frac{\partial }{\partial r}=0,\frac{\partial }{\partial \varphi }=0$$

Therefore, we have the simplified equations,

(Equation 8) $$\nabla^{2}p=\frac{1}{r^{2}\;\sin\;\theta }\frac{\partial }{\partial \theta }\left(\sin\;\theta \frac{\partial p}{\partial \theta }\right)$$

and

$$\frac{\partial^{2}p}{\partial t^{2}}-\frac{c^{2}}{r^{2}}\left(\frac{\partial^{2}p}{\partial \theta^{2}}+\cot\;\theta \frac{\partial p}{\partial \theta }\right)=0$$

Equation 8 has singular points at θ=0° and θ=180° (Stone and Goldbart, 2009). This is typical for problems with a point source, such as the electric field induced by an electric particle (Feynman et al., 2010) at an idealized (geometric) point. Considering the spherical waves propagating on the “surface” of the Earth (presumably in the upper troposphere and perhaps also including the lower stratosphere), the wave would be dependent on the variable

ξ=rθ−Ut

where U is the celerity (or phase speed of the shockwaves). With the above variable, we can obtain the dispersion relationship of the waves as

$$U^{2}=c^{2}-i\frac{c^{2}}{r}\cot\;\theta$$

or

(Equation 9) $$U=c\sqrt{1-i\frac{1}{r}\cot\;\theta }=c\alpha$$

Here $i=\sqrt{-1}$. The above equation is the dispersion relationship for the large scale shockwaves propagating in the atmosphere as spherical waves.

#### Instrument and data

The sensors used in this study were the Bosch Sensortec BME280 digital humidity, pressure, and temperature sensors (Bosch, 2021) for measurements in the air. The pressure sensors had a range of 300hPa to 1100hPa with an RMS noise of 0.2 Pa. The sampling frequency was set at 1 Hz. The sensor was integrated by the author with a microprocessor, a UBLOX NEO-6M Global Positioning System (GPS) module, and an SD card for data recording. A total of two sensor packages, one run by an AC power supply and one powered by solar panel charged batteries, are deployed at two locations in Baton Rouge, Louisiana, U.S.A. The two sites are separated by 9.6 km. The first package was deployed at the Ridge station or Site 1 (Table 1, 91.0912° W, 30.3695° N); while the second was deployed at the Russell station or Site 2 (Table 1), 9.6 km northwest of the first at (91.1795°W, 30.4116°N). The sensors were inside a ventilated weather-shielded box filled with replaceable desiccant and deployed with a free connection to air. The data (Li, 2022a) were validated by air pressure measurements from the closest ASOS weather station at the Baton Rouge airport ∼ about 14.2 km north of Site 2 and 19.5 km north-northwest of Site 1.

The first dataset from the Ridge station (Site 1) has 3 s ensemble recording intervals. The sensor has been collecting air-pressure data for more than 10 years. The second dataset from the Russell stations (Site 2) has 21 s ensemble recording intervals. This sensor has been collecting data for 8 years. In this study, the data in January 2022 are used.

In addition to these data, we also used ASOS data from 151 weather stations recording data at 1 min intervals and 38 weather stations (Tables 1, 2, 3, and 4) recording data at 5 min intervals throughout the contiguous U.S., Alaska, Hawaii, tropical Pacific (Guam and Saipan Islands), and tropical Atlantic Oceans (Puerto Rico). There are comparable data around the world, e.g., data from 40 stations in Britain and Ireland (Burt, 2022) were used in analysing the shockwave generated by the volcano eruption of the Hunga Tonga-Hunga Ha’apai.

The time series of high-resolution air pressure data from Sites 1 and 2 are first QA/QCed by excluding invalid data (less than 0.5% of the total data points) followed by interpolation to fill the data points where invalid data are excluded.

#### Distance and propagation speed estimate

Based on observations from the geostationary satellite GEOS-17 (World Bank, 2022), shockwaves were generated following the eruption on 15 January 2022 and the waves propagated around the globe in the form of (surficial) spherical waves (SW). Conceptually, if we assume that the Earth is a perfect sphere, the SWs should radiate outward with the volcano as a pole on the globe (P in Figure 1). The radius of the SWs increases to 90° from point P (Figure 1B). As the SWs propagate toward the antipole (point A in Figure 1B), the radius decreases until it reaches the antipole of the volcano, after which they propagate back as return SWs with the original antipole (point A in Figure 1B) of the volcano position becoming the pole and point P the antipole for the return waves. Since the volcano is at (20.55°S, 175.385°W), it is situated near the International Date Line and the antipole is at (20.55°N, 4.615°E), which is in southern Algeria. Because of the spherical propagation of the waves, the analysis must calculate the distance between the source location at P and the air pressure observation stations.

Given a location, the spherical shockwaves have two possible routes (Figure 1) on the great circle determined by the location of the source P and location of signal reception S (Station): the first is the shortest spherical arc *L*_1_ = $\hat{PS}$while the second is the longer arc going through the antipole A, *L*_2_ = $\hat{PAS}$. If the shockwave is strong enough to allow multiple passes around the world, and if we denote the circumference of the Earth as *L*, the distances of passes are: first pass: *L*_1_; second pass: *L*_2_; third pass: *L* + *L*_1_; fourth pass: *L* + *L*_2_; fifth pass: 2*L* + *L*_1_; sixth pass: 2*L* + *L*_2_ … In general, the formula is

$$L=\left(k-1\right)L+\{\begin{array}{l} L_{1}\\ L_{2} \end{array},\;\left(k=1,2,\ldots \right)$$

Using the timing of the signal, we can estimate the wave propagation speed to be $v=\frac{L}{\Delta t}$. For example, for the first pass:

$$v_{1}=\frac{L_{1}}{T_{1}-T}\;$$

where, $T_{1}$ is the time of the first arrival of the signal and T is the time of the eruption. Likewise, we can estimate the wave propagation speed for the second pass:

$$v_{2}=\frac{L_{2}}{T_{2}-T}\;$$

in which $T_{2}$ is the time of the second arrival of the shockwaves. The speed values $v_{1}$ and $v_{2}$ for these two passes should be consistent (about the same). At this point, it should be noted that it is a little tricky to choose the exact start time of the eruption for the computation of the shockwave propagation speed. There are several different times reported as discussed earlier, ranging from 0402 UTC to 0415 UTC. In a numerical model study (Amores et al., 2022), the eruption time was defined at 0430 UTC. There were even several smaller eruptions starting from Dec. 20, 2021, which did not seem to have caused any global scale shockwaves. Satellite images (Bachmeier, 2022) suggest that the start time of the catastrophic eruption was before 0410 UTC on 15 January but reached maximum around 0415 UTC and lasted for about 1 h (World Bank, 2022). To allow a globally propagating wave, the wave energy must have exceeded certain threshold. Although we do not know what the threshold value is, in the computation, we reasonably assume that the peak arrival of the shockwave corresponds to the peak eruption. Therefore, we use the reported time of the maximum explosion (World Bank, 2022) as the start time *T* in the above equations. This, however, may still have some uncertainties. An error estimate with some sensitivity computation is discussed in the last subsection of the method section.

#### Spherical distance

The distance between Hunga Tonga-Hunga Ha’apai underwater volcano and a given weather station is computed assuming that the Earth is a perfect sphere. Since the Earth is close to an ellipsoid, the error introduced using this assumption is approximately 0.3% for distance computation (e.g., Vermeille, 2002). With this in mind, and given that the longitude and latitude of two points ($P_{1}$ and $P_{2}$) on the surface of Earth are

$$P_{1}=\left(\lambda_{1},\varphi_{1}\right),P_{2}=\left(\lambda_{2},\varphi_{2}\right)$$

where $\lambda_{1}$ and $\lambda_{2}$ are the longitudes and $\varphi_{1}$ and $\varphi_{2}$ are the latitudes of the two points, respectively. Spherical trigonometry (Bronshtein et al., 2015) gives the following cosine equation for the shortest distance on a sphere between two points:

$$\cos\;a=\sin\;\varphi_{1}\;\sin\;\varphi_{2}+\cos\;\varphi_{1}\;\cos\;\varphi_{2}\;\cos\left(\lambda_{2}-\lambda_{1}\right)\;$$

Here, a is the arc of the great circle on Earth’s surface between the two points. This leads to

$$a=\arccos\left(\sin\;\varphi_{1}\;\sin\;\varphi_{2}+\cos\;\varphi_{1}\;\cos\;\varphi_{2}\;\cos\left(\lambda_{2}-\lambda_{1}\right)\right)$$

Given that Earth’s average radius r=6371 km, the distance between the two points on the sphere is

$$L_{1}=\bar{P_{1}P_{2}}=ra$$

The Hunga Tonga-Hunga Ha’apai underwater volcano is located at (20.55°S, 175.385°W). The distance between the volcano and the first (second) sensor location or Site 1 (Site 2) is 10,627 (10,621) km (Table 1). The distances between the volcano and the additional 189 weather stations from the contiguous U.S., Alaska, Hawaii, tropical Pacific (Guam and Saipan Islands), and tropical Atlantic Oceans (Puerto Rico) are also computed in this way (Tables 1, 2, 3, and 4), with the closest station being that in the central Pacific (Hawaii, ∼5000 km) and the farthest station being that at Frenchville, Maine (12,941 km).

For the second route, the distance the waves travel is

$$L_{2}=2\pi r-ra$$

### Quantification and statistical analysis

#### Error estimate

There are sources of error in the computation of the shockwave speed. These include errors for the distance and arrival time, assuming the eruption time is accurate. Because the Earth is closer to an ellipsoid, its radius at the equator is approximately 6378 km, whereas the polar radius is approximately 6357 km. The use of an average radius of 6371 km can thus introduce an error on the order of ∼*e*_*d*_ = 0.3%. The error in arrival time is determined by the sampling interval, assuming that the time from the GPS at each station is negligible. Because the arrival time of the first signal is on the order of 10 h (Table 2), a sampling interval of 3 s leads to a relative error of ∼ *e*_*t*1_ = 0.008%. For data with 21 s intervals, the relative error is ∼ *e*_*t2*_ = 0.06%. For the lowest resolution data, the sampling interval is 5-min, leading to a relative error of the arrival time of ∼ *e*_*t3*_ = 0.8%. The error of the propagation speed can be obtained from the errors of the distance and arrival time (e.g., Bevington and Robinson, 2003)

$$e_{v}=e_{d}+e_{t}$$

where *e*_*d*_ and *e*_*t*_ are the relative errors of the distance calculation and signal arrival time estimate, respectively. Using this formula and the above estimate, the maximum error of shockwave speed estimate is *e*_*v*_ = *e*_*d*_ + *e*_*t*_ ∼ 1.1%. For the second signal, the relative errors are smaller; thus, the above relative error estimate provided the upper limit.

If the eruption time is inaccurate, the error may increase. In our computation, the eruption time is selected at the reported maximum eruption time. If this time is inaccurate, how will the computation result change? To answer this question, we have experimented with the adjustment of the start time from 04:14:45 UTC to to 0402 UTC. Satellite images suggest that the eruption started right before 0410 UTC so the use of 0402 UTC is an extreme scenario. The results show that the average speed for all the stations would be 302.6 ± 4.5 m/s and 306.7 ± 1.4 m/s for the first and second signals through Routes 1 and 2, respectively. This tells us two things: (1) the change in such a extreme possibility does not change the propagations speed greatly (it changes 7/309–2.3%); and (2) this scenario is unlikely because the two speeds have much larger difference (∼4 m/s vs. the original 0.5 m/s). The larger value of speed (306.7 m/s) for the second signal indicates that the assumed eruption time is probably too early (so that the first signal appears to have arrived with a “slower” speed while the second signal with a “faster” speed). If the eruption time is selected at 0410 UTC, the speed is 306.0 ± 4.5 and 307.9 ± 1.4 m/s for the first two signals. The difference of the two speeds is ∼1.9 m/s, which is between 4 m/s and 0.5 m/s. The conclusion is: the use of the reported maximum eruption time (0414 UTC) gives the smallest difference (0.5 m/s) and is a reasonable choice.

## Data Availability

• The original data of the high-resolution air pressure measured by the author using his lab-made sensor package is available at the DOI listed in the key resources table. • The low-resolution data from weather stations are open source from NOAA and the author does not re-post third party’s data but can provide them individually if requested. • Any additional information about the paper and MATLAB scripts for the analysis can be available from the lead contact upon request.
